# Experiences of caregivers desiring to refuse life-prolonging treatment for their elderly parents at the end of life

**DOI:** 10.1080/17482631.2019.1632110

**Published:** 2019-06-18

**Authors:** Yuki Maura, Mariko Yamamoto, Tomoko Tamaki, Ryo Odachi, Mikiko Ito, Yuri Kitamura, Tomotaka Sobue

**Affiliations:** a Department of Nursing, Faculty of Nursing and Rehabilitation, Konan Women’s University, Kobe, Hyogo, Japan; b Division of Health Sciences, Graduate School of Medicine, Osaka University, Suita, Osaka, Japan; c Department of Nursing, School of Nursing, Mukogawa Women’s University, Nishinomiya, Hyogo, Japan; d Department of Nursing and Laboratory Science, Graduate School of Medicine, Yamaguchi University, Yamaguchi, Japan; e Department of Nursing, Shiga University of Medical Science, Otsu, Shiga, Japan; f Division of Environmental Medicine and Population Sciences, Department of Social and Environmental Medicine, Graduate School of Medicine, Osaka University, Osaka, Japan

**Keywords:** Family caregiver, elderly, life-prolonging treatment, end-of-life care, qualitative analysis

## Abstract

**Purpose**: This study aimed to clarify the experiences of caregivers desiring to refuse life-prolonging treatment for their elderly parents at the end of life.

**Methods**: A semi-structured interview was performed for four family caregivers who wanted to refuse life-prolonging treatment suggested by the physicians.

**Results**: In this study, four caregivers who refused life-prolonging treatment suggested by the physicians for their elderly parents completed semi-structured interviews. The obtained data were analyzed in relation to the theme “Experiences of caregivers who desire to refuse life-prolonging treatment for their elderly parents at the end of life.” As a result, 38 subcategories and 12 categories were extracted.

**Conclusions**: Participants in this study initially had a negative view of life-prolonging treatment. However, they agonized over the decision when they received conflicting advice from the physicians. The participants indicated that physicians’ advice and attitudes complicated their decisions to reject life-prolonging treatment for their elderly parents.

## Introduction

As of October 2017, Japan’s aging rate was 27.7%, far exceeding that in other countries (Cabinet Office, Government of Japan, 2018). Consequently, life-prolonging treatment for elderly persons has attracted substantial attention. A previous opinion survey found that most people wish to die naturally without life-prolonging medical treatment at the end of life (Ministry of Health, Labor and Welfare, 2014). A high number of respondents desired antibiotics and intravenous treatment for pneumonia and intravenous feeding once the ability for oral consumption was lost. However, 57%–78% of respondents did not desire central vein, nasal, or gastric feeding, artificial respiration, or cardiopulmonary resuscitation. These results suggest that when patients have no chance of recovery because of age, people do not desire life-prolonging medical treatment. Although most people do not desire life-prolonging treatment, approximately 70% of elderly patients at the end of life have lost decision-making capacity (Silveira, Kim, & Langa, 2010). In Japan, few people provide written instructions regarding their intention to receive life-prolonging treatment; their families are generally responsible for such decisions.

Domestic studies on surrogate decision-making by family members are increasing (Aoki, 2014; Kato & Takeda, 2017). The main themes include decisions on whether to accept artificial hydration and nutrition (AHN), receive treatment at medical facilities or at home, and accept resuscitation. Most domestic studies on surrogate decision-making for end-of-life care for elderly persons targeted family caregivers who selected gastrostomy, focusing on the psychological state of the families and the decision-making process (Aiba & Oizumi, 2011; Kato & Hara, 2012; Kato, Kajitani, & Ito et al., 2011; Kurata & Yamashita, 2011; Makino, Kinouchi, & Minoura et al., 2013; Mizuoka & Fujinami, 2014; Yamamoto, Morikawa, & Erami et al., 2014). However, few studies have examined the experiences of families who refuse life-prolonging treatment for elderly relatives (Kato & Takeda, 2017). In such studies conducted in other countries, the experiences of families in medical environments, mainly emergency medicine and acute treatment, were analyzed with less focus on elderly patients (Meeker & Jezewski, 2008).

Guidelines that define tube feeding as inappropriate for patients with severe dementia have been developed in other countries (Aita, 2012a). Tube feeding does not improve nutritional status, prevent aspiration pneumonitis, or improve QOL and vital signs in patients with severe dementia (Gillick, 2000; Suzuki, 2012; Tomas, Christmas, & Travis, 1999). One controversial life-prolonging treatment is percutaneous endoscopic gastrostomy (PEG). It was developed in 1979 for pediatric patients who have oral intake difficulties. However, in the 2000s, PEG spread rapidly in Japan to treat elderly patients who cannot sustain life through their oral intake. AHN such as PEG is provided to elderly patients who are beyond recovering from senility or frail patients with dementia who have communication difficulties. Therefore, PEG faced ethical and moral dilemmas. No strict guidelines support withholding treatment for elderly patients at the end-of-life stage and the physicians tend to apply life-prolonging treatment included PEG to avoid psychological distress and legal liability (Aita K, Miyata H, Takahashi M, Kai I. 2008; Aita, 2011).

Further discussion is needed to obtain a consensus regarding refusal of life-prolonging treatment such as AHN for elderly patients in Japan. Therefore, the actual decision-making situation must be clarified. This study aims to clarify the experiences of caregivers who refused life-prolonging treatment for their elderly patients.

## Materials and methods

### Participants and data collection

The participants of this study were family members who provided end-of-life care to elderly persons older than 80 years in the past 1–2 years. For these elderly persons, life-prolonging treatment (tube feeding, parenteral nutrition, respirator) was introduced or suggested by the physicians.

We called several support groups and associations of family caregivers to introduce us to research participants. Inclusion and exclusion criteria are related to the experience of withholding or withdrawing life-prolonging treatment for elderly persons. In this study, we researched four family caregivers who clearly indicated their family’s decision to the physicians regarding withholding or withdrawing life-prolonging treatment for elderly persons.

### Design and data analysis

The examination method was a semi-structured interview administered in Japanese. The survey period was November 2015 to March 2016, and the interview duration was 60–90 min. The questions assessed the experiences of caregivers over the end-of-life period through death of their elderly care receivers. The questions focused on issues such as the decision to select life-prolonging treatment and healthcare support.

To clarify the experiences of caregivers desiring to refuse life-prolonging treatment for elderly relatives, a qualitative descriptive approach was selected (Greg, 2016; Sandelowski, 2000). From the interview contents recorded with the approval of the participants, anonymized records were created as study data.

The analyzed items were events that made the participants aware of the possibility of life-prolonging treatment as well as thoughts and emotions associated with the decision. The responses were categorized and encoded. During the analysis, the researchers returned to the written record as needed, and the codes were examined and interpreted by repeatedly reading the content. Data were collected by comparing the similarities and differences in the codes for each subject, and the level of abstraction was raised and assumed as subcategories. Next, the similarities and differences of the subcategories were compared, the level of abstraction was raised, and categories were created in the same manner. The data for each subject were analyzed by multiple researchers, and bias in the results and distortion in their interpretation were examined until agreement was obtained among the researchers.

### Ethical considerations

This study was approved by the Ethical Review Board of Osaka University Hospital (Date of approval: 19 October 2015, Approval number: 15201). The participants were informed that their participation in the study was voluntary and that they would not receive any disadvantage by participating, forgoing participation, or withdrawing. Informed consent was obtained from all participants. Moreover, the participants were informed that they could withdraw from the study at any time after the interview, and a consent withdrawal statement was given to each subject. Further, the participants were informed that the research would be presented at research conferences or published in research journals, and each subject agreed to the publicization of the data.

## Results

In this study, four caregivers who refused life-prolonging treatment suggested by the physicians for their elderly parents completed semi-structured interviews (Table I). The obtained data were analyzed in relation to the theme “Experiences of caregivers who desire to refuse life-prolonging treatment for their elderly parents at the end of life.” As a result, 38 subcategories and 12 categories were extracted (Table II).

**Table I.** Summary of the participants.

Subject	Age group (years)	Elderly person (age at the time of death, years)	Place of treatment (at the time of death) Causes of death	Dementia (communication)	Written instructions regarding medical care	Accepted medical care Rejected medical care
A	70–79	Mother (102)	At home (At home) Heart failure	Yes (Difficult)	Unknown	Intravenous nutrition Subcutaneous infusion Oral administration treatment Gastric feeding
B	60–69	Mother (91)	Nursing home (hospital) Infectious disease	Yes (Difficult)	Unknown	Fracture surgery Intravenous nutrition Intravenous Hyperalimentation Gastric feeding Respirator
C	60–69	Mother-in-law (86)	Special elderly nursing home (Special elderly nursing home) Pneumonia	Yes (Difficult)	Unknown	Gastric feeding Intravenous Hyperalimentation
D	60–69	Mother (91)	Nursing home (Psychiatric hospital) Heart failure	Yes (Difficult)	Written will	Changing battery of pacemaker Fracture surgery Intravenous nutrition Intravenous Hyperalimentation Gastric feeding

**Table II.** Experiences of caregivers desiring to refuse life-prolonging treatment for their elderly parents at the end of life.

Category	Subcategory
Life-prolonging treatment viewed as an action that deprives the parent of pleasure and joy	Prolong the pain of the parent Artificial hydration and nutrition will not restore the parent’s health Life-prolonging treatment deprives the parent of pleasure and humanity Meaning of life is the ability to consume food
Knowledge of the limit of care that can be provided by family	Never-ending care Home health care aggravates dementia symptom Motherly qualities were lost to dementia
Satisfaction with sufficiently provided care	Wish to obtain satisfaction from medical care Spent the entire life with the parent I was the only sibling who cared about the parent
Acceptance that death cannot be avoided	Parent continually weakening Awareness that death is imminent Understand recovery is impossible
Limited possibility of recovery conveyed by the physicians	Oral intake is impossible Impossible to continue parenteral nutrition Gastric feeding and central vein feeding are essential for life support
Limits of recovery revised by the physicians	Regarding mobility following bone fracture surgery Possibility of recovery is not 0% Consciousness is maintained with a respirator
Acceptance of medical care that is not life-prolonging	Provide treatment to revive swallowing function Provide artificial hydration and nutrition through methods other than gastric feeding
The meaning of “natural death” was revised by the physicians	Not providing treatment will cause the parent to starve Not providing treatment will cause the parent to experience pain Physicians stress the pain of the parent Physicians blame caregivers for not accepting that death is imminent Resuscitation is attempted following a sudden change in the parent’s condition until the family arrives
Refusal of treatment led to ejection from medical facilities	The parent can enter the facilities if artificial hydration and nutrition is administered Facility admission was denied unless curative treatment was attempted Hospitalization cannot continue without accepting treatment
Request support regarding the decision to accept life-prolonging care	Learn the burden or risk to the parent based on the literature and discussions with people close to the parent Confirm the desire of the parent from a written will or other information People close to the parent convey their beliefs that the parent would refuse life-prolonging treatment
Acceptance of death	Do not introduce new medical care even when the condition changes Spend time together to ensure a peaceful death Accepting that death is imminent
Affirming the decision to forgo life-prolonging treatment	“Natural death” was painless for the parent Refused further treatment suggested by physicians Respected the humanity of the parent

### Life-prolonging treatment viewed as an action that deprives the parent of pleasure and joy

Caregivers valued oral food consumption, and thus, they had negative feelings regarding providing their elderly parents AHN via oral or nasal feeding tubes. Moreover, they regarded life-prolonging treatment as an action that deprives their parents of pleasure and joy and prevented them from enjoying independent lives. Their thought process was based on the assumption that their parents would not recover to their prior state even if life-prolonging treatment were provided.

“People reach the end of natural life when they can no longer feed themselves … it is unnatural to force them to continue living through life-prolonging treatment. (snip) Even if they remain alive, there is no pleasure because they are confined to bed. It is good if they express satisfaction in receiving care, but they will never return to their previous state … ” (A)

“I believe peaceful death is very important. I don’t think it’s a good idea to force people to continue to live, which takes away their dignity. If they undergo gastric feeding, they would just spend their days confined to bed. I doubt if you could call it ‘living.’ I wouldn’t want it for me or my mother.” (D)

### Knowledge of the limit of care that can be provided by family

The families experienced large burdens in caring for their relatives, and they were aware of the limits of care that they could provide. This knowledge also led to recognition of the limits of recovery for elderly persons and life prolongation, which influenced the decision to refuse life-prolonging therapy. One caregiver attempted treatment to restore the swallowing function of his parent, who was unable to swallow and was confined to bed because of severe dementia. However, because he found that his parent was unwilling to attempt oral feeding, he found himself exhausted and reconsidered the meaning and limits of care.

“I doubted if it would be good for my mother to live that way … I found myself exhausted, and I felt we had to end it. The helper tried to encourage me, saying things such as ‘Let’s get a new Japanese long-living record!’ However, I was not interested in such a record. (snip) When I was providing Chinese medicine to restore my mother’s swallowing ability, her facial expression confirmed that she did not like its bitter taste. It made me start thinking about what I could next do for my parent. I was tired, and I stopped using the medicine, saying, ‘Why don’t we end this, mom?’” (A)

One caregiver disagreed with her brothers’ opinion that it was unnecessary to change the batteries of her mother’s pacemaker because of her age and severity of dementia. However, as the mother’s symptoms worsened and her delusional behaviors increased, it was hard for the caregiver to accept that her mother’s “maternity” was gradually lost. Her thoughts about providing subsequent treatment to her mother changed and gradually strengthened her determination to reject life-prolonging treatment.

“She slept more and struggled to stay awake, and she was delusional … when I visited her, she said things such as, ‘Go back, now!’ or ‘You’ll be attacked by someone!’ I didn’t want to hear such words anymore.” (D)

### Satisfaction with sufficiently provided care

The families wished to explore every option based on their experiences with care. When they became aware of their parents’ impending death, they did not desire life-prolonging treatment but instead wanted to affirmatively accept death. Meanwhile, some caregivers selected active treatment to ensure that they were satisfied with the care they provided their parents and avoid regret after their deaths.

“I was not prepared for my parent’s death … I requested treatment to revive her swallowing function for my own satisfaction so I could feel like, ‘I’ve done everything I could and it must be enough. I didn’t want to have regrets such as ‘I should’ve done this or that.’ I realized I forced my parent to live for my own reasons … ” (A)

### Acceptance that death cannot be avoided

The physical and psychological weakening of the elderly parents at the end-of-life stage gradually progressed. Their families were aware that death was imminent as they perceived daily changes in their parents’ condition. The families worked to accept that death was an unavoidable reality.

“I felt something different. She was sleeping more, and she looked blank even when I talked to her. I felt something different.” (C)

“She didn’t eat much, and her condition got worse after the bone fracture. She was gradually getting weaker. Her kidney was also damaged, and she was unable to discharge urine. So, I gradually prepared for her death … ” (D)

### Limited possibility of recovery conveyed by the physicians

The physicians informed the families of the limited possibility of recovery and likelihood of death before confirming whether life-prolonging treatment was desired. When it became difficult for patients to consume food orally, a gastric feeding tube and intravenous therapy were needed. Moreover, when it became difficult for patients to receive intravenous therapy, middle cardiac vein infusion was needed. The treatment was progressively adjusted until death, and families had to decide on multiple occasions whether to accept life-prolonging treatment.

“The infusion leaked because the blood vessels were already damaged. They tried arms, legs, and other areas, but none worked. Eventually, the doctors suggested gastric tube feeding and said she would pass away if the infusion could not be performed.” (A)

### Limits of recovery revised by the physicians

The families were aware of the limited possibility of recovery following discussions with the physicians. Even when the patients were unable to eat and their conditions gradually deteriorated, surgical treatment for bone fracture was performed. The families did not believe that their parents would regain the ability to walk and considered the surgery pointless. However, the doctors devised a standard treatment plan including rehabilitation after surgery to restore mobility in the patients.

“I was told that she needed to be hospitalized and undergo surgery when she broke a bone. She was unable to move. She said she didn’t feel pain as long as she didn’t have to move, so I told the doctor that I did not want surgery. However, the doctor said he would perform surgery even if the patient were 100 years old … so I thought there was no other choice but to accept the surgery. He said he would perform rehabilitation, but she was unable to eat … the doctor told me after about a week that rehabilitation wasn’t possible.” (B)

The families were informed that death was imminent, but at the same time, they were told that recovery was possible if treatment was performed. Although the families prepared for the deaths of their parents, they were confused by the information presented by the physicians.

“After central vein feeding, the doctors recommended gastric feeding, treatment for sudden low blood pressure, and artificial respiration. I asked them if my mother would recover, and they said, ‘the possibility is not zero.’ I told him that I didn’t understand the purpose of using respirators, and then he took me to one ward in the hospital. What I saw there was … you know there were about eight patients in beds and monitors placed by them. I thought ‘Oh my.’ He said patients with respirators were not necessarily unconscious. (snip) He asked an old woman, one of the patients there, to raise her hand, and the old woman with a respirator raised her hand slightly. The doctor said that we could see that our mom would still be alive even if she were in such a condition … ” (B)

### Acceptance of medical care that is not life-prolonging

Although most of the families were not in favor of life-prolonging treatment, their understanding of its meaning varied. Whereas they strongly refused gastric feeding, they were less resistant to other forms of AHN such as parenteral feeding, central vein feeding, and subcutaneous infusion, as well as various pharmacotherapies to revive swallowing function. For the families, there was no contradiction between refusing life-prolonging treatment and selecting medical care that did not prolong life.

“I thought it would have been nice to let my mother remain alive if she were able to eat. Therefore, I considered the swallowing treatment. (snip) Even if it was useless and the doctors and hospital disagreed … they provided the treatment when we requested it.”(A)

### The meaning of “natural death” was revised by the physicians

Forgoing life-prolonging treatment was based on the caregivers’ desire to allow their parents to die naturally without pain. However, the physicians informed the families that their parents would experience pain if allowed to die naturally.

“I asked the doctor if my parent would die naturally if intravenous hyperalimentation was not performed … she was confined to bed and unable to move. She was also feeling tired, so … I told the doctor that I didn’t want to receive further treatment. The doctor then said that she would starve to death without treatment. I asked him if it would be painful for her, and he said starving to death without being given food would be painful for anybody.” (B)

“I told the doctor that I was not going to change the batteries of my mother’s pacemaker. I created a written agreement with my brothers. (snip) The doctor said that she would suffer severely when the batteries died. So … we decided to change them because we didn’t want her to suffer … I was given lots of confusing information … but I bet she wouldn’t have suffered that much, right?” (D)

The families who selected natural death expressed their desire to avoid life-prolonging treatment and resuscitation. However, when the parents were near death and the families were absent, the physicians attempted resuscitation to allow the families to be with their parents at the time of death against the families’ will.

“When I saw the doctor giving cardiac massage to my mother, I thought he was giving a respirator, so I said she didn’t need further treatment. However, the doctor said he would do his best until all of the family members arrived. (snip) I repeatedly told the doctor not to provide treatment anymore. But he said he would try a little more … ” (B)

### Refusal of treatment led to ejection from medical facilities

The families understood that it would be difficult to keep their parents hospitalized if they refused treatment. However, nursing facilities, which aim to provide care to elderly people during the end-of-life stage, considered sudden changes, such as death in emergency or due to acute deterioration, in elderly patients as risks and attempted to avoid such patients due to shortages of labor and professional staff. Therefore, regardless of the desires of the patients and families, life-prolonging treatment was forbidden, and invasive surgical treatment was recommended. In some cases, the contract with facilities was canceled when such requests by the facilities were rejected. The families were worried about their parents losing access to end-of-life care at medical facilities.

“I told the doctor that I didn’t request surgery for her bone fracture because it would only be painful. Then I was told that they couldn’t admit my mother if I refuse their recommendation for surgery (the parent cannot go back to the facilities). I also was told by the doctor at the hospital where my parent was hospitalized that they couldn’t accept her if I didn’t accept the surgery.” (D)

“I was persuaded by the facility staff members that … if I don’t change the batteries of the pacemaker, they won’t be able to respond when her heart stopped. They said they wouldn’t be able to care for my parent if I didn’t change the batteries. At the time when my mother entered the facilities, I was told that I would be able to care for my parent until the very end … but the policy was changed without notice … ” (D)

### Request support regarding the decision to accept life-prolonging care

When the parents’ desires were unknown, their families experienced significant conflict regarding the decision to accept life-prolonging treatment. Before making the decision, the families collected information from the literature and experienced persons to obtain support for the idea that life-prolonging treatment should not be selected in an effort to prevent pain.

“I read many books about gastric feeding and had my husband read them, too. I learned about it and drew my conclusion. (snip) I learned the risks of gastric feeding, such as … providing too many nutrients at the end of life could cause heart damage … ” (C)

Meanwhile, the families were hesitant to forgo life-prolonging treatment even when their parents had clearly stated instructions. Even when their parents were at the end-of-life stage and exhibited difficulty communicating their desires, their families wished to understand their will at that time.

“When I took my mother to the hospital, I tried to converse with her through writing because she was unable to talk. I wrote, ‘Do you wish to die?’ Well … somebody said to me later that it was terrible to ask such a question … I was asked by the doctor if I wished to receive life-prolonging treatment. I asked her ‘Yes’ or ‘No’ in writing, and she pointed to ‘No.’ (snip) I was really encouraged, and I told the doctor not to give life-prolonging treatment. Her response really encouraged me.” (D)

The families who hesitated to decide as their parents’ desires were unknown were encouraged by affirmative words provided by the physicians and people close to their parents. People close to the families were all reliable persons who knew the patients and their families well, and the families considered that their advice represented their parents’ thoughts.

“After central vein feeding, I really hated telling the doctor not to give further treatment. When I met a doctor whom I knew near my house, he asked me if my mother was alright. I told him I was worrying about what I should do next. He said, ‘If I were your mother, I wouldn’t wish further treatment. She must have received treatment enough … ’ Another story is … I met an old man I knew at the market. He is in his 80s. I told him about my concerns, and he said ‘I wouldn’t want life-prolonging treatment. Your mom is such a strong woman. So, I believe she does not want such a treatment’.” (B)

### Acceptance of death

During the process before the patients’ deaths, various medical options were presented to the families. It was reminded to the families that refusing life-prolonging treatment meant that death was imminent. This caused hesitation among the families in making the decision. The families used various strategies to strengthen their determination regarding the decision.

“My present feeling, my feeling when I was living with my mother, my mother’s feeling when she got married to my father … I was so confused … but I needed to calm myself, you know … to summarize those feelings. So, I wrote to the doctor saying we are not going to request further treatment.” (B)

The families who overcame the conflict were able to accept their last moments with their parents with peaceful feelings.

“I was encouraged by my parent’s declaration of her intentions, and I was determined not to provide life-prolonging treatment anymore. I was with my parent until the last moment, saying things such as ‘peaceful death is coming soon mom’ … so it was good.” (D)

### Affirming the decision to forgo life-prolonging treatment

The families declared their intentions to forgo life-prolonging treatment while giving meaning to the choice. Moreover, after their parents’ deaths, the caregivers affirmed that they had made the correct choice.

“It really was a peaceful death, so I was convinced it was the right decision. Now I know natural death is just peaceful … so it was the right decision for us. However, I could never say that I can recommend that others refuse life-prolonging treatment, you know. The decisions will differ for each family. In our case, refusing life-prolonging treatment was the right choice.” (C)

Although some families had no choice but to accept the life-prolonging treatment proposed by doctors, they aimed to convince themselves that the right decision had been made.

“Looking back now, I unwillingly chose central vein feeding, but maybe … it was good that I could spend time with my mother until her death. It was the right decision to refuse further treatment proposed by the doctor.” (B)

## Discussion

### Meaning of refusing life-prolonging treatment by family

The participants of this study gave meaning to their own decisions to refuse life-prolonging treatment for their parents. Moreover, they rejected life-prolonging treatments such as gastric feeding, regarding it as an action that deprived their parents of pleasure and joy in their final moments. As mentioned previously, few reports have examined the experiences of families who refuse life-prolonging treatments such as AHN for elderly relatives. However, various reports have evaluated families who selected PEG. Aiba et al. reported that families who chose PEG for elderly relatives with severe dementia wished to prolong their relatives’ lives (Aiba & Oizumi, 2011). They were told by doctors that PEG was safe and that its management was easy, and although they selected the treatment based on doctors’ advice, they were hesitant because their parents’ wishes were unknown. Further, Kato et al. reported that families wished to avoid death and decided to allow their relatives to undergo PEG based on their self-determination to assume responsibility as a substitute, believing that PEG was safe and that their parents would have supported the decision (Kato & Hara, 2012). The study participants initially had negative views of life-prolonging treatment and expressed these without hesitation. However, they agonized over the decision if they received conflicting advice from the physicians. To resolve this conflict, they needed to accept that death could not be avoided while being satisfied that sufficient care was provided or understanding the limits of care that could be provided.

Some parents had written instructions refusing life-prolonging treatment. However, they did not regard certain medical treatments, such as infusion for rehydration or antibiotics for pneumonia as life-prolonging care, and we presumed that they would accept such therapy. Even if they lacked written instructions rejecting life-prolonging treatment, they possibly did not understand what life-prolonging treatment included. Families must understand the definition of life-prolonging treatment and the reality and characteristics of the end-of-life stage when selecting care for their parents. In this regard, families’ experiences described in this study should be fully used to understand the difficulties faced by family caregivers and develop potential solutions.

### Problems with physicians’ support of decisions

The study participants indicated that the advice and attitudes of the physicians complicated their decisions to reject life-prolonging treatment for their elderly parents. The families indicated that their understanding of the meanings of “life” and “death” were changed by this advice. Contrarily, the advice and attitudes of medical staff did not change throughout the process, and they recommended standard medical care even if the elderly patient was at the end-of-life stage. The discrepancy between doctors’ advice and families’ beliefs regarding the limits of medical intervention is notable (Ando, 2012). In terms of medical care and technical involvement, healthy people are considered the standard, and diseases and disorders are recognized as problems that fall short of this standard. This viewpoint does not mesh with the reality of people with diseases and disorders or those receiving end-of-life care (Ando, 2012). The value of end-of-life care for elderly people should be transformed from a medical viewpoint that seeks recovery from diseases and prolongation of life to care that does not deny the likelihood of worsening and death.

In Japan, when oral food and water intake becomes impossible, AHN such as PEG is provided for elderly patients with dementia or those nearing the end of their lives. A survey of doctors specializing in geriatrics found that approximately one-third of respondents selected gastric and nasal tube feeding for elderly people with severe dementia (Aita, 2012b). The participants in this study revealed that the physicians used negative expressions such as “death by starvation” when they refused life-prolonging treatment for their elderly parents. Moreover, fears among the physicians of legal responsibility and ethical conflicts over decisions such as reducing, withdrawing, or refusing AHN have been noted (Aita, 2011). Further education of the physicians is needed to ensure that the guidelines for end-of-life care can be fully used (The Japan Geriatrics Society, 2012).

Furthermore, a problem of the healthcare system is families’ fears of losing access to medical care for refusing treatment. Although the families recognized that their parents were at the end-of-life stage, they could not secure medical care for their parents in some cases, which prompted them to accept unwanted medical treatment to ensure that their parents could enter or remain in the hospital. Thus, an environment in which families can care for their parents without accepting unwanted treatment is vital.

## Limitations

Participants in this study believed that they did not receive appropriate support from the physicians regarding their decision-making. Therefore, it is possible that they joined the study with various feelings of distress and regret. Therefore, we should consider possible self-selection bias. Additionally, this study involved few participants and cannot suggest a large variety of families’ experiences. It is still insufficient to understand family caregivers’ decision-making experiences. Moreover, various forms of family relationships should be analyzed because the relationship between the elderly patient and their family is thought to be important factor in surrogate decision-making.
